# Análise da Custo-Efetividade da Angiotomografia Coronariana no SUS, em Comparação com Outros Métodos Não Invasivos na Suspeita de DAC Estável

**DOI:** 10.36660/abc.20201050

**Published:** 2022-01-11

**Authors:** Patricia Bastos do Carmo, Carlos Alberto da Silva Magliano, Helena Cramer Veiga Rey, Gabriel C. Camargo, Luís Filipe Lannes Trocado, Ilan Gottlieb

**Affiliations:** 1 Instituto Nacional de Cardiologia Rio de Janeiro RJ Brasil Instituto Nacional de Cardiologia , Rio de Janeiro , RJ – Brasil; 2 Casa de Saúde São José Rio de Janeiro RJ Brasil Casa de Saúde São José , Rio de Janeiro , RJ – Brasil

**Keywords:** Doença das Coronárias, Angina Estável, Análise Custo-Benefício, Técnicas de Diagnóstico Cardiovascular

## Abstract

**Fundamento:**

Atualmente o sistema de saúde público brasileiro (SUS) não contempla a angiotomografia de coronárias.

**Objetivos:**

Ranquear sob a perspectiva do SUS, a custo-efetividade de estratégias diagnósticas combinando teste ergométrico, cintilografia miocárdica, ecocardiograma por estresse e angiotomografia de coronárias para o diagnóstico de doença arterial coronariana em uma coorte hipotética de pacientes com probabilidade pré-teste intermediária.

**Métodos:**

Análise de custo-efetividade por meio de árvore de decisão. Foram analisados a relação de custo-efetividade incremental e o benefício líquido em saúde das estratégias diagnósticas, com a adoção de múltiplos limiares de disposição a pagar entre 0,05 e 1 PIB per capita por diagnóstico correto. Nos casos de testes sequenciais, um segundo teste confirmatório era realizado quando o primeiro fosse positivo.

**Resultados:**

Após exclusão das estratégias diagnósticas dominadas ou com dominância estendida, a fronteira de eficiência foi composta por três estratégias: teste ergométrico, teste ergométrico seguido de ecocardiograma de estresse, e ecocardiograma de estresse seguido de angiotomografia de coronárias, sendo esta última a estratégia mais custo-efetiva. Pelo critério do benefício líquido, o ranqueamento das estratégias mais custo-efetivas variou conforme a disposição a pagar.

**Conclusão:**

Utilizando conceitos atuais de avaliação de tecnologias em saúde, este estudo fornece um ranqueamento para a tomada de decisão sobre qual estratégia diagnóstica utilizar, em uma população com risco pré-teste intermediário para DAC. Com estimativa factível de custos para a ATC, o impacto da inclusão desta ao rol do arsenal diagnóstico representaria uma estratégia custo-efetiva na maioria dos cenários avaliados nas variações de disposição a pagar.

## Introdução

A doença cardiovascular foi causa de 17,7 milhões de óbitos em 2015, representando 31% de todas as mortes em nível global. Desses, estima-se que 7,4 milhões ocorram devido à doença coronariana (DAC). ^1 , 2^ No Brasil, de acordo com última atualização dos indicadores de saúde, foram registrados aproximadamente 490.000 óbitos por DAC no período de 2007 a 2011. ^3^ Estima-se que a prevalência de angina leve e angina moderada à grave na população brasileira seja respectivamente de 7,6% e 4,2%, ^4^ e os custos relacionados a doenças cardiovasculares crescem à medida que a população envelhece, sendo estimado em 2015, para o Brasil, um total de R$ 37,1 bilhões de reais, aproximadamente 0,7% do produto interno bruto (PIB). ^5^

A cineangiocoronariografia (CAT) é o “padrão-ouro” para diagnóstico de DAC, porém é exame invasivo e associado a complicações. ^6 , 7^ Idealmente, testes diagnósticos não invasivos deveriam selecionar quais pacientes seriam encaminhados para confirmação diagnóstica invasiva, mas a estratégia atual é falha, como demonstrado em um grande registro de 398.978 pacientes encaminhados à CAT, dos quais apenas 37% apresentaram DAC obstrutiva, apesar de testes não invasivos terem sido realizados em 84% dos pacientes (em sua maioria funcionais). ^8^ Um estudo brasileiro corrobora esses achados, no qual 61% dos pacientes com testes funcionais com critérios de alto risco não apresentaram DAC obstrutiva. ^9^ Novos testes diagnósticos com maior acurácia ou estratégias diagnósticas sequenciais têm potencial de reduzir os erros diagnósticos e o número de CAT desnecessárias.

Identificar a estratégia diagnóstica para DAC obstrutiva mais custo-efetiva pode trazer benefícios clínicos e econômicos para o SUS. Hoje, além da CAT, os testes diagnósticos para DAC disponíveis no SUS são: cintilografia miocárdica (CM), ecocardiograma de estresse (ECO) e teste ergométrico (TE). A angiotomografia coronariana (ATC) é um exame ainda não incorporado no SUS, embora apresente alta acurácia diagnóstica, quando comparada aos demais. ^10^

O objetivo deste estudo é ranquear a custo-efetividade das diferentes estratégias diagnósticas de DAC, considerando os testes não invasivos disponíveis no SUS, e a ATC, testando variados limiares de disposição a pagar, para uma população pré-estabelecida de probabilidade pré-teste intermediária de 30%, dentro da perspectiva do SUS.

## Métodos

A razão de custo-efetividade incremental (RCEI) tem sido rotineiramente utilizada por agências de avaliação de tecnologia em saúde em todo o mundo para resumir os resultados das avaliações econômicas e estabelecer a custo-efetividade das tecnologias. Entretanto, uma nova metodologia de avaliação de custo-efetividade foi proposta: o benefício líquido, que pode ser monetário, em inglês, *net monetary benefit* (NMB), ou benefício em saúde, em inglês, *net health benefit* (NHB). ^14^ Essa última metodologia tem vantagens sobre a RCEI, por não necessitar de um comparador base para estimativa de ganhos e custos incrementais e por ser mais fácil calcular. A eficácia ou “benefício” de cada estratégia pode ser medida em diferentes formas, como anos de vida salvos, ou pelo número de diagnósticos corretos obtidos com uma estratégia diagnóstica. Estima-se, com base em uma disposição a pagar pré-estabelecida, o “lucro” obtido com a intervenção. Por exemplo, se um decisor está disposto a pagar R$ 10 mil reais por ano de vida salvo, uma tecnologia que aumentasse em 5 anos a sobrevida “valeria” R$ 50 mil reais. Caso o preço da intervenção fosse inferior a R$ 50 mil reais, ela seria benéfica. Para um valor hipotético de R$ 30 mil reais, tal intervenção estaria fornecendo um NMB de R$ 20 mil reais (5 x R$ 10 mil – R$ 30 mil). Da mesma maneira, considerando a mesma disposição a pagar, esperamos um ganho mínimo de 3 anos de vida com tal investimento (30 mil/R$ 10 mil), mas, como ele fornece 5 anos de sobrevida, teremos um NHB de 2 anos. Quanto maior o ganho monetário ou em saúde, maior a custo-efetividade daquela tecnologia ou estratégia diagnóstica, pois sua incorporação trará economia e ganhos em saúde.

A custo-efetividade dos testes diagnósticos para DAC obstrutiva (ATC, CM, ECO e TE) foi avaliada usando uma combinação de 11 estratégias diagnósticas e o impacto sobre uma coorte hipotética de 1.000 indivíduos com 30% de prevalência de DAC (probabilidade intermediária). Um teste negativo representava o fim da pesquisa. Nos casos em que a estratégia diagnóstica envolvesse testes sequenciais, um segundo teste confirmatório era realizado apenas caso o primeiro teste fosse positivo. A soma de testes verdadeiros negativos (teste negativo em pacientes sem DAC >50%) com testes verdadeiros positivos (testes positivos em pacientes com DAC >50%) representou o total de diagnósticos corretos.

Para definição da estratégia mais custo-efetiva, foram adotadas duas análises: a fronteira de eficiência, com base na RCEI e o NHB. Por não haver um limiar de custo-efetividade estabelecido no Brasil, todas as tecnologias não dominadas ou sem dominação estendida foram apresentadas por meio de uma fronteira de eficiência. Com as estratégias ranqueadas de acordo com seus custos ou benefícios, a estratégia dominada será simplesmente aquela menos eficaz e mais cara.

A segunda etapa de análise pelo RCEI envolve a identificação de estratégias com dominância estendida. As estratégias não dominadas foram ranqueadas por ordem crescente de custos, e a RCEI, calculada comparando os custos e efetividade incrementais relativamente à estratégia menos onerosa precedente. As estratégias menos eficazes e com uma RCEI mais elevada foram consideradas não custo-efetivas por dominância estendida.

A estratégia mais custo-efetiva, por definição, é aquela que apresenta a maior RCEI, dentro do limiar de disposição a pagar estabelecido pelo tomador de decisão. No caso do NHB, a estratégia mais custo-efetiva será aquela que trouxer o maior ganho líquido em número de diagnósticos corretos, de acordo com cada limiar de disposição a pagar. Para ambas as análises, foi necessário estimar os custos e a efetividade (quantidade de testes corretamente diagnosticados) das diferentes estratégias.

As estratégias mais custo-efetivas foram também ranqueadas de acordo com a variação de probabilidades pré-teste entre 10% e 60% em diferentes limiares de disposição a pagar por um diagnóstico correto (tabela em anexo e disponível no Mendeley Data), tendo como base o valor do PIB per capita brasileiro. Todos os cálculos foram realizados no Excel®.

### Custos

A estimativa de custos ( tabela 1 ) foi realizada por meio da abordagem *top down* , e o custo de cada estratégia foi baseado no custo unitário de cada teste. Para os testes disponíveis no SUS, os custos foram obtidos pelo SIGTAP - Sistema de Gerenciamento da Tabela e Procedimentos, Medicamentos e OPM do SUS. ^3^ Para a ATC, foi utilizada a abordagem de microcusteio *botom up* para quantificação dos recursos necessários para sua realização (apêndice 1).

**Tabela 1:** – Custos dos testes diagnósticos em ordem crescente

Teste	Custo unitário (SIGTAP)
Teste ergométrico	R$ 30,00
Ecocardiograma de estresse	R$ 165,00
Angiotomografia coronariana	R$ 452,05
Cintilografia miocárdica estresse e repouso	R$ 791,59

*Valores extraídos da tabela SIGTAP no ano de 2020 ^3^*

### Efetividade

A acurácia de cada teste foi estimada com base em uma revisão da literatura realizada em 20/09/2019 com uma busca por meta-análises sobre a acurácia dos testes diagnósticos nas bases de dados MEDLINE, The Cochrane Library e Lilacs, sem restrição ao idioma. Os estudos foram selecionados de forma independente por dois revisores (P.B. e L.T.) e as discordâncias resolvidas por consenso. Se ao final da seleção de estudos houvesse mais de 1 artigo selecionado, o estudo com melhor avaliação de qualidade pelo AMSTAR ^15^ seria utilizado. A estratégia de busca e o fluxograma de seleção das evidências estão disponíveis respectivamente nos apêndices 2 e 3.

### Análise de Sensibilidade

Para avaliar o impacto das incertezas dos valores das variáveis inseridas no modelo, foi realizada a análise de sensibilidade determinística. Foram utilizados os intervalos de confiança ou interquartil como valores máximos e mínimos de cada informação contida no modelo a partir de revisão de literatura, evidenciados na tabela 2 .

**Tabela 2:** – Parâmetros e valores adotados no modelo de RCEI para estratégias diagnósticas de DAC

Parâmetro	Estimativa pontual	Limite Inferior	Limite Superior	Referência
Sensibilidade TE	0,80	0,48	0,85	Banerjee et al.,2012 ^16^
Especificidade TE	0,63	0,63	0,88	Banerjee et al.,2012 ^16^
Sensibilidade ECO	0,81	0,70	0,87	Banerjee et al.,2012 ^16^
Especificidade ECO	0,84	0,73	0,94	Banerjee et al.,2012 ^16^
Sensibilidade CM	0,88	0,88	0,89	Jaarsma et al., 2012 ^17^
Especificidade CM	0,61	0,59	0,62	Jaarsma et al., 2012 ^17^
Sensibilidade ATC	0,93	0,93	0,94	Haase et al., 2019 ^18^
Especificidade ATC	0,84	0,84	0,85	Haase et al., 2019 ^18^

*Teste Ergométrico e EcoEstresse: (Banerjee, Newman, Van Den Bruel, & Heneghan, 2012); Cintilografia (Jaarsma et al., 2012); ATC (Haase et al., 2019). TE: teste ergométrico; CM: cintilografia miocárdica; ATC: angiotomografia de coronárias; ECO: ecocardiograma por estresse.*

### Considerações éticas

Não foram realizadas pesquisas em seres humanos, nem utilizados dados confidenciais, institucionais ou pessoais. Toda a pesquisa é baseada em dados de estudos publicados em base de dados eletrônicas. Este projeto recebeu o seguinte parecer do CEP: “Trata-se de uma pesquisa de revisão sistemática da literatura que não necessita de avaliação pelo CEP”. Número do parecer: 2.421.181.

## Resultados

Na análise pela RCEI, dentre as 11 estratégias diagnósticas, foram identificadas 7 estratégias dominadas, ou seja, estratégias com maior custo e menor número de diagnósticos corretos ( tabela 3 ).

**Tabela 3:** – Projeção das estratégias em 1000 pacientes em ordem de custo, com o número de diagnósticos corretos e identificação das dominadas

Estratégia	Custo	Diagnóstico correto	Observação
TE	R$ 30.000,00	681	
TE + ECO	R$ 112.335,00	853	
ECO	R$ 165.000,00	831	Dominada
TE + ATC	R$ 255.572,95	884	
ECO + ATC	R$ 325.477,75	909	
ATC	R$ 452.050,00	871	Dominada
ATC + TE	R$ 463.732,00	884	Dominada
ATC + ECO	R$ 516.301,00	909	Dominada
ATC + CM	R$ 760.295,15	904	Dominada
CM	R$ 791.590,00	691	Dominada
CM + ATC	R$ 1.034.340,85	904	Dominada

*TE: teste ergométrico; CM: cintilografia miocárdica; ATC: angiotomografia de coronárias; ECO: ecocardiograma por estresse. Ano 2020 como referência para os valores apresentados.*

As quatro estratégias não dominadas foram ordenadas por ordem crescente de custos, e identificou-se que a estratégia TE + ATC era menos efetiva (menor número de diagnósticos corretos) e com maior RCEI do que a estratégia ECO + ATC ( tabela 4 ), sendo, portanto, considerada não custo-efetiva (dominância estendida).

**Tabela 4:** – Identificação de dominância estendida nas estratégias diagnósticas para DAC não dominadas

Estratégia	Custo	Diagnóstico correto	RCEI	
TE	R$ 30.000,00	681,00	NA	
TE + ECO	R$ 112.335,00	852,96	R$ 478,80	
TE + ATC	R$ 255.572,95	883,75	R$ 4.651,19	Dominância estendida
ECO + ATC	R$ 325.477,75	909,49	R$ 2.716,44	

*TE: teste ergométrico; CM: cintilografia miocárdica; ATC: angiotomografia de coronárias; ECO: ecocardiograma por estresse. Ano 2020 como referência para os valores apresentados; RCEI: razão de custo-efetividade incremental.*

**Figura 1:**
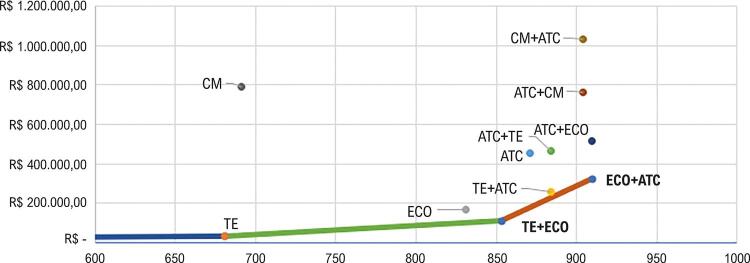
– Fronteira de eficiência das estratégias diagnósticas para doença coronariana (custos e número de diagnósticos corretos por mil indivíduos). TE: teste ergométrico; CM: cintilografia miocárdica; ATC: angiotomografia de coronárias; ECO: ecocardiograma por estresse.

Assim, a fronteira de eficiência foi construída com base nas três estratégias mais custo-efetivas, TE, TE+ECO e ECO+ATC ( figura 1 ).

**Figura 2:**
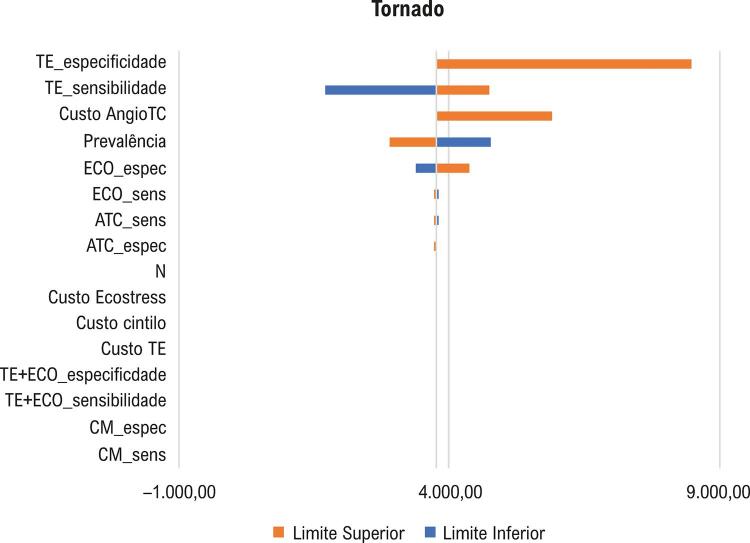
– Diagrama de Tornado – impacto nos valores de RCEI de cada parâmetro avaliado isoladamente em seus limites inferior e superior.

Baseado na análise de sensibilidade, evidenciada no gráfico de Tornado ( figura 2 ), os parâmetros com maior impacto nos resultados foram a sensibilidade e especificidade do teste ergométrico, o custo da ATC e a prevalência de DAC.

O ranqueamento pelo NHB permite avaliar todas as estratégias, sem a necessidade de excluir estratégias dominadas ou com dominância estendida. A tabela 5 apresenta o ranqueamento das estratégias mais custo efetivas de acordo com a variação de probabilidades pré-teste entre 10% e 60% em diferentes limiares de disposição a pagar por um diagnóstico correto, tendo como base o valor do PIB per capita brasileiro, que segundo última atualização do IBGE 2017 é de R$ 31.833,50. ^19^

**Tabela 5:** – Ranqueamento das estratégias diagnósticas por limiar de disposição a pagar por diagnóstico correto de acordo com o critério de benefício líquido, em relação às diferentes probabilidades pré-teste

	0,05 PIB pc	0,1 PIB pc	0,2 PIB pc	0,3 PIB pc	0,4 PIB pc	0,5 PIB pc	1 PIB pc
10%	TE + ECO	TE + ECO	ECO + ATC	ECO + ATC	ECO + ATC	ECO + ATC	ECO + ATC
20%	TE + ECO	TE + ECO	ECO + ATC	ECO + ATC	ECO + ATC	ECO + ATC	ECO + ATC
30%	TE + ECO	ATC + TE	ECO + ATC	ECO + ATC	ECO + ATC	ECO + ATC	ECO + ATC
40%	TE + ECO	ATC + TE	ECO + ATC	ECO + ATC	ECO + ATC	ECO + ATC	ECO + ATC
50%	ECO	ATC + TE	ATC	ATC	ATC	ATC	ATC
60%	ECO	ECO	ATC	ATC	ATC	ATC	ATC

*ATC: angiotomografia coronariana; CM: cintilografia miocárdica; ECO: ecocardiograma de estresse; TE: teste ergométrico; PIB: produto interno bruto.*

## Discussão

A estratégia ECO + ATC apresenta a melhor taxa de diagnóstico correto (909,49), ou seja, a maior efetividade; e, portanto, maior certeza no direcionamento clínico dos pacientes, seja para a coronariografia, seja para afastar o diagnóstico. A definição das melhores estratégias de investigação de DAC obstrutiva resulta em maior certeza diagnóstica, minimizando o número de falso-negativos (perda do diagnóstico), assim como os falso-positivos (reduzindo número de cateterismos “brancos” e suas complicações). ^20 , 21^ Erros diagnósticos nesse cenário estão associados a exames invasivos desnecessários, além de poderem levar a complicações como infarto agudo do miocárdio e morte devido à falta de tratamento adequado em uma doença de alta mortalidade. ^1 , 2 , 20^ A acurácia dos testes diagnósticos deve ser analisada à luz de seus custos, principalmente quando consideramos uma nova tecnologia em um sistema de saúde financiado publicamente, como o SUS.

A razão de custo-efetividade incremental (RCEI) tem sido usada por agências de avaliação de tecnologia em saúde em todo o mundo para resumir os resultados de avaliações econômicas de intervenções em saúde. Mesmo em países como o Brasil, onde não existe um limiar explícito de RCEI para tomada de decisão, seu impacto nas decisões é muito relevante. No entanto, medidas sumárias alternativas baseadas no conceito de benefício líquido vêm sendo apresentadas e este é o primeiro estudo a avaliar o custo-benefício das estratégias diagnósticas para DAC por meio de uma análise de benefício líquido em saúde.

Existem distinções importantes entre o RCEI e o NHB. O RCEI necessita de comparação entre duas estratégias, independentemente do número total de estratégias avaliadas. No NHB, as medidas de benefício líquido são calculadas para cada estratégia individualmente, ou seja, elimina-se a necessidade de comparação por pares e da eliminação de estratégias dominadas. É necessário um limiar estabelecido de disposição a pagar para calcular medidas de benefício líquido, mas não é necessário para calcular a RCEI, embora sem um limiar de custo-efetividade sua interpretação fique limitada. Não há um limiar de custo-efetividade estabelecido no Brasil no processo de incorporação de tecnologias. Neste estudo, a estratégia mais custo-efetiva, de acordo com a fronteira de eficiência, foi a combinação de ecocardiograma de estresse seguido de angiotomografia de coronárias, dado que é a estratégia não dominada com maior valor de RCEI e dentro de um limiar.

O critério de NHB permite ranquear todas as estratégias de forma a ajudar o tomador de decisão a escolher qual estratégia seguir, com base na disponibilidade dos exames/profissionais, orçamento e disposição a pagar por diagnóstico correto. A ATC, apesar de ser o único exame não incorporado hoje ao SUS, é o exame mais prevalente entre as estratégias mais custo-efetivas, apenas não ocupando o primeiro lugar quando a disposição a pagar é inferior a 0,2 PIB per capita por diagnóstico correto. Isoladamente, desconsiderando exames sequenciais, o exame mais custo-efetivo é o ecocardiograma de estresse, até o limiar de 0,1 PIB per capita por diagnóstico correto, sendo superado pela ATC nos limiares superiores.

Ao variarmos em diferentes probabilidades pré-teste (10% a 60%) encontramos a ATC como teste mais custo efetivo (combinado ou não a outros métodos) em 79% dos cenários analisados, o que está em acordo com estudos de custo-efetividade da ATC realizados em países desenvolvidos ^20 - 23^ além das recentes atualizações das diretrizes do Reino Unido ( *National Institute for Heath and Care Excellence* ) de 2017 ^22 , 24^ e da diretriz da sociedade europeia (ESC). ^25^ Atualizada em 2019, tal diretriz determina que a ATC possa ser usada como primeiro exame na avaliação de sintomas sugestivos de DAC obstrutiva, em alternativa aos exames funcionais de imagem. No Reino Unido, que possui um sistema de saúde financiado com recursos públicos (assim como o SUS no Brasil) baseado em análises de custo-efetividade para a sua realidade, foi optado pela recomendação da ATC como primeiro exame, em substituição aos exames funcionais. ^22 , 24^ Essa decisão deve ser tomada baseada na realidade de cada país e cada sistema de saúde, sendo o objetivo do presente estudo aprofundar o entendimento das estratégias diagnósticas de dor torácica na realidade do SUS.

A escolha da estratégia diagnóstica deve levar em consideração não apenas a custo-efetividade para o achado da obstrução coronariana, mas também os desfechos clínicos. Grandes estudos randomizados como o PROMISE e SCOT HEART trouxeram informações principalmente sobre o prognóstico daqueles pacientes que iniciavam a investigação para DAC com a angiotomografia de coronárias. Houve maior certeza no diagnóstico e, com isso, maior introdução de terapias medicamentosas preventivas, considerando uma possibilidade real na redução de eventos como infarto a longo prazo, além de documentar menor número de coronariografias sem doença obstrutiva nesse grupo de pacientes. ^26 , 27^ Nesse contexto é importante ressaltar que o grande estudo randomizado ISCHEMIA não demostrou redução de morte ou infarto no braço dos exames funcionais. ^28^ Em contrapartida, a avaliação anatômica permite o diagnóstico de aterosclerose e direciona melhor tratamento clínico, com possibilidade na redução de infarto e morte. ^26 , 27 , 29^ A última diretriz da sociedade brasileira de cardiologia (SBC) de 2014, recomenda iniciar a investigação de DAC obstrutiva com exames funcionais, seguidos por ATC caso tais exames sejam inconclusivos ou contraindicados. ^30^

Dentre os métodos funcionais, a CM é um dos testes mais usados no Brasil e no mundo para diagnóstico de DAC, ^31 , 32^ sendo estimado que no SUS aproximadamente 54% dos exames eletivos de medicina nuclear sejam de perfusão miocárdica. ^32^ No entanto, as estratégias que incluíram CM foram dominadas em nosso estudo, sendo mais caras e menos efetivas.

As estratégias TE + ECO e TE + ATC apresentam percentuais de falso positivo semelhantes de 4% (tabela 2:2 em anexo), já o percentual de falsos negativos varia de forma considerável entre as estratégias. Por exemplo, a estratégia TE + ECO apresenta taxa de falso negativo de 1,3%, e a estratégia TE + ATC, com taxa de falso negativo de 0,4% (três vezes menor do que TE + ECO), dessa forma apresentando menos diagnósticos errados. Apesar de ter dominância estendida na fronteira de eficiência, observamos na análise do NHB que a estratégia TE + ATC perde para TE + ECO apenas na margem de 0,05 a 0,1 PIB per capita (tabela NHB em anexo). Características econômicas assimétricas entre regiões e cidades no Brasil fazem com que a disponibilidade de equipamentos e de mão de obra qualificada seja heterogênea. Por exemplo, no ano de 2019, dentre as quase seis milhões de tomografias realizadas no SUS, 51% concentraram-se na região Sudeste e menos de 6%, na região Norte. ^33^ O ranqueamento das opções diagnósticas apresentado neste trabalho poderá auxiliar os decisores combinando dados locais de infraestrutura, disposição a pagar e acurácia diagnóstica. A ATC é um exame menos difundido e com maquinário mais caro que o ECO, o que possivelmente a limita como primeiro exame em cenários com menor orçamento, em que o ideal seria iniciar com um exame de menor custo e complexidade, sendo os resultados positivos referenciados para exames mais caros e de maior complexidade. Sendo o teste ergométrico exame mais difundido que o ECO, há de se considerar que a estratégia TE + ATC possa ser mais exequível no sistema de saúde público brasileiro do que a estratégia ECO + ATC, apesar de pequena queda da efetividade da iniciada pelo TE comparada com a iniciada pelo ECO. Analisamos primariamente pacientes em probabilidade pré-teste de 30%, por ser essa a prevalência de doença usualmente encontrada nos laboratórios diagnósticos ambulatoriais (considerada moderada-baixa). Após a realização dos testes de sensibilidade, os resultados de custo-efetividade não revelam mudanças substanciais quando variamos a probabilidade pré-teste. Além disso, foram ranqueadas as probabilidades pré-teste entre 10 e 60% de acordo com a disposição a pagar, sendo a ATC não encontrada como opção custo efetiva apenas quando consideramos o valor de 0,05 PIB per capita por diagnóstico correto.

Dentre as limitações desse trabalho, encontram-se divergências entre os artigos encontrados, principalmente em relação à definição de DAC obstrutiva, em que os estudos sobre ATC e CM definem como obstrução coronariana acima de 50% à coronariografia, e o estudo que inclui teste ergométrico e ecoestresse inclui artigos com referência de DAC obstrutiva acima de 50% ou 70%. Para minimizar possíveis vieses, foi utilizada estratégia de revisão sistemática além da utilização de meta-análises e análise da qualidade dos artigos. Um recente estudo de custo-efetividade com dados do SUS apresenta como principal limitação a determinação do valor da ATC como o custo pago pelo SUS por uma tomografia simples de tórax, extrapolando dados do sistema de saúde suplementar. ^34^ Nosso trabalho tenta se aproximar ao valor real de custos da ATC (aproximadamente 3 vezes o valor da TC de tórax), uma vez que esse valor influencia majoritariamente a avaliação das estratégias comparativas. Por fim, a acurácia dos testes varia com a qualidade do equipamento e dos profissionais responsáveis. Futuros estudos poderão atestar o impacto da adoção deste fluxograma de decisão pelo seguimento prospectivo computando dados clínicos e econômicos dos resultados de mundo real.

## Conclusão

Em cenários como o brasileiro, de restrição orçamentária e de heterogeneidade na oferta de testes diagnósticos, identificar estratégias custo-efetivas poderá orientar gestores e tomadores de decisão em saúde a gerir seus recursos de maneira mais eficiente. Utilizando conceitos atuais de avaliação de tecnologias em saúde, este estudo fornece um ranqueamento para a tomada de decisão sobre qual estratégia diagnóstica utilizar, em uma população com risco pré-teste intermediário para DAC. Adotando-se uma estimativa factível de custos para a ATC, conclui-se que o impacto da inclusão desta ao rol do arsenal diagnóstico representaria uma estratégia custo-efetiva na maioria dos cenários avaliados com amplas variações na disposição a pagar.
