# Supplementary material for: Análise da Custo-Efetividade da Angiotomografia Coronariana no SUS, em Comparação com Outros Métodos Não Invasivos na Suspeita de DAC Estável
Source: Arq Bras Cardiol. 2022 Jan 11;118(3):578–85. [Article in Portuguese] doi: 10.36660/abc.20201050 (PMC8959029; doi:10.36660/abc.20201050)
Supplement: Translation

# Translation: Cost-Effectiveness Analysis of CCTA in SUS, as Compared to Other Non-Invasive Imaging Modalities in Suspected Obstructive CAD

**Authors:** Patricia Bastos do Carmo, Carlos Alberto da Silva Magliano, Helena Cramer Veiga Rey, Gabriel C. Camargo, Luís Filipe Lannes Trocado, Ilan Gottlieb

**Affiliations:** 1 Brazil Instituto Nacional de Cardiologia, Rio de Janeiro, RJ - Brazil; 2 Brazil Casa de Saúde São José, Rio de Janeiro, RJ – Brazil

**Keywords:** Coronary Disease, Stable Angina, Cost-Benefit Analysis, Diagnostic Techniques, Cardiovascular

## Abstract

**Background:**

The Brazilian public health system does not include computed tomography angiography (CTA).

**Objective:**

Rank, according to the Brazilian public health system, the cost-effectiveness of different strategies for the diagnosis of coronary artery disease (CAD), combining exercise tests (ET), myocardial scintigraphy (MS), stress echocardiography (SE), and CTA in a hypothetical intermediate pre-test probability cohort of patients.

**Methods:**

This study implemented a cost-effectiveness analysis through a decision tree. The incremental cost-effectiveness ratio (ICER) and net benefit were analyzed by adopting multiple thresholds of willingness to pay, from 0.05 to 1 GDP per capita per correct diagnosis. In sequential tests, a second confirmatory test was performed only when the first was positive.

**Results:**

After excluding dominated or extended dominance diagnostic strategies, the efficiency frontier consisted of three strategies: ET, ET followed by SE, and SE followed by CTA, the last being the most cost-effective strategy. Through the net benefit, the ranking of the most cost-effective strategies varied according to willingness to pay.

**Conclusions:**

Using current concepts of health technology assessment, this study provides a ranking for decision-making concerning which diagnostic strategy to use in a population with an intermediate pre-test risk for CAD. With a feasible cost estimate adopted for CTA, the impact of including this to the list of the diagnostic arsenal would represent a cost-effective strategy in most of the evaluated scenarios with broad variations in the willingness to pay.

## Introduction

Cardiovascular disease was the cause of 17.7 million deaths in 2015, representing 31% of all deaths worldwide. Of these, it is estimated that 7.4 million occur due to coronary artery disease (CAD). ^1 , 2^ In Brazil, according to most recent health indicators, approximately 490,000 deaths were reported from 2007 to 2011. ^3^ It is estimated that the prevalence of light angina and mild to severe angina in the Brazilian population is, respectively, 7.6% and 4.2%, ^4^ and the costs related to cardiovascular diseases grow as the population ages, estimated, in 2015, for Brazil, at a total of R$37.1 billion, or approximately 0.7% of the gross domestic product (GDP). ^5^

Coronary angiography (CA) is the “gold standard” for the diagnosis of CAD; however, it is an invasive exam and associated with complications. ^6 , 7^ Ideally, non-invasive tests should select which patients should be referred for invasive diagnostic confirmation, but the current strategy is flawed, as demonstrated in a massive record of 398,978 patients referred for the CA, of which only 37% presented obstructive CAD, even though non-invasive tests were conducted in 85% of the patients (mostly functional). ^8^ One Brazilian study corroborates these findings, in which 61% of the patients with functional tests with high-risk criteria did not present obstructive CAD. ^9^ New diagnostic tests with greater accuracy or sequential diagnostic strategies have the potential to reduce the diagnostic errors and the unnecessary number of CAs.

Identifying the most cost-effective diagnostic strategy for obstructive CAD can bring clinical and economic benefits for the Brazilian Unified Health System (SUS, in Portuguese). Today, in addition to the CA, the diagnostic tests for CAD available at SUS are: myocardial scintigraphy (MS), stress echocardiography (SE), and exercise tests (ET). The computed tomography angiography (CTA) is an exam that is still not included in SUS, although it does present high-accuracy diagnoses, when compared to the other exams. ^10^

The present study aims to rank the cost-effectiveness of the different CAD diagnostic strategies, considering the non-invasive tests available at SUS, and the CTA, testing varied thresholds of the willingness to pay for a pre-defined 30% intermediate pre-test probability population within the realm of SUS.

## Methods

The incremental cost-effectiveness ratio (ICER) has been routinely used by health technology assessment agencies worldwide to summarize the results of economic assessments and establish the cost-effectiveness of technologies. However, a new methodology of cost-effectiveness assessment was proposed: the *net monetary benefit* (NMB), or the *net health benefit* (NHB). ^14^ This latter methodology has advantages over the ICER, as it does not require a base comparison to estimate the gains and incremental costs, and as it is easier to calculate. The efficiency or “benefit” of each strategy can be measured in different forms, such as years of lives saved, or by the number of correct diagnoses obtained with a diagnostic strategy. Based on a pre-defined willingness to pay, the “profit” obtained with the intervention is estimated. For example, if a decision-maker is willing to pay R$10,000 per year per saved life, a technology that increases survival in 5 years would be “worth” R$50,000. If the interventional price were less than R$50,000, it would be beneficial. For a hypothetical value of R$30,000, such an intervention would be providing an NMB of R$20,000 (5 x R$10,000– R$30,000). Likewise, considering the same willingness to pay, we would expect a minimum gain of 3 years of life with such an investment (30,000/R$10,000), but, as it provides 5 years of survival, we will have an NHB of 2 years. The greater the monetary or health gain, the greater the cost-effectiveness of that technology or diagnostic strategy, as its incorporation will bring savings and gains in health.

The cost-effectiveness of the diagnostic tests for obstructive CAD (CTA, MS, SE, and ET) was assessed using a combination of 11 diagnostic strategies and the impact on a hypothetical cohort of 1,000 individuals with a 30% prevalence of CAD (intermediate probability). A negative test represented the end of the study. In cases in which the diagnostic strategy involved sequential tests, a second confirmatory test was carried out only if the first test was positive. The sum of true negative tests (negative test in patients without CAD>50%) with true positive tests (positive tests in patients with CAD>50%) represented the total correct diagnoses.

To define the most cost-effective strategy, two analyses were adopted: the efficiency frontier, one based on the ICER and one based on the NHB. As there is no cost-effectiveness threshold established in Brazil, all of the technologies that were not dominated or without extended dominance were presented through an efficiency frontier. With the strategies ranked according to their costs or benefits, the dominated strategy will simply be that which is less efficient and more expensive.

The second stage of the ICER analysis involves the identification of strategies with extended dominance. The undominated strategies were ranked in an ascending order of costs, and the ICER was calculated by comparing the costs and incremental effectiveness related to the prior least costly strategy. The less efficient strategies and those with a lower ICER were considered non-cost-effective by extended dominance.

The most cost-effective strategy, by definition, is that which presents the highest ICER, within the threshold of the willingness to pay established by the decision-maker. In the case of the NHB, the most cost-effective strategy will be that which brings the highest net gain in number of correct diagnoses, according to each threshold of willingness to pay. For both analyses, it was necessary to estimate the costs and the effectiveness (quantity of correctly diagnosed tests) of the different strategies.

The most cost-effective strategies were also ranked according to the variation of pre-test probabilities between 10% and 60% in different thresholds of willingness to pay for a correct diagnosis (table attached here and available at Mendeley Data), the Brazilian GDP per capita using as a base value. All of the calculations were done in Excel®.

### Costs

The estimation of costs ( Table 1 ) was created by means of a *top-down* approach, and the cost of each strategy was based on the unit cost of each test. For the tests available at SUS, the costs were obtained through SIGTAP (Management System for the Table of Procedures, Medications and OPM) from SUS. ^3^ For CTA, the *bottom-up* micro-cost approach was used to quantify the resources necessary for its fulfillment (appendix 1).

**Table 1:** – Costs of diagnostic tests in ascending order

Test	Unit cost (SIGTAP)
Exercise test	R$ 30.00
Stress echocardiogram	R$ 165.00
Computed tomography angiography	R$ 452.05
Stress and at-rest myocardial scintigraphy	R$ 791.59

*Values extracted from the SIGTAP table in 2020 ^3^*

### Effectiveness

The accuracy of each test was estimated based on a literature review carried out on September 20, 2019, with a search for meta-analyses about the accuracy of diagnostic tests in the MEDLINE, The Cochrane Library, Lilacs databases, with no restrictions on languages. The studies were selected separately by two reviewers (P.B. and L.T.); disagreements were resolved by consensus. If at the end of the selection of the studies there was more than one article selected, the study with the best quality evaluation, according to AMSTAR. ^15^ was used. The search strategy and the flowchart of the selection of evidence is available, respectively, in appendixes 2 and 3.

### Sensitivity analysis

To evaluate the impact of uncertainties of the values inserted in the model, a deterministic sensitivity analysis was performed. The confidence and interquartile intervals were used as the maximum and minimum values of each piece of information contained in the model as of the literature review, as shown in Table 2 .

**Table 2:** – Parameters and values adopted in the ICER model for CAD diagnostic strategies

Parameter	Accuracy	Lower Limit	Upper Limit	Reference
ET Sensitivity	0.80	0.48	0.85	Banerjee et al.,2012 ^16^
ET Specificity	0.63	0,63	0.88	Banerjee et al.,2012 ^16^
SE Sensitivity	0.81	0.70	0.87	Banerjee et al.,2012 ^16^
SE Specificity	0.84	0.73	0.94	Banerjee et al.,2012 ^16^
MS Sensitivity	0.88	0.88	0.89	Jaarsma et al., 2012 ^17^
MS Specificity	0.61	0.59	0.62	Jaarsma et al., 2012 ^17^
CTA Sensitivity	0.93	0.93	0.94	Haase et al., 2019 ^18^
CTA Specificity	0.84	0.84	0.85	Haase et al., 2019 ^18^

*Exercise Test and Stress Echocardiogram: (Banerjee, Newman, Van Den Bruel, & Heneghan, 2012); Scintigraphy (Jaarsma et al., 2012); CTA (Haase et al., 2019). ET: exercise test; MS: myocardial scintigraphy; CTA: computed tomography angiography; SE: stress echocardiogram.*

### Ethical Consideration

No studies were performed on human beings, nor were confidential, institutional, or personal data used. The entire study is based on data published in electronic databases. This project received the following report from the Research Ethics Committee (REC): “This study is a systematic review of the literature that does not require an assessment on the part of the REC”; logged under report number: 2.421.181.

## Results

In the ICER analysis, among the 11 diagnosed strategies, 7 dominated strategies were identified, that is, strategies with a higher cost and a lower number of correct diagnoses ( Table 3 ).

**Table 3:** – Projection of the strategies in 1,000 patients in order of cost, with the number of correct diagnoses and the identification of the dominated strategies

Strategy	Cost	Correct diagnosis	Observation
ET	R$ 30,000.00	681	
ET + SE	R$ 112,335.00	853	
SE	R$ 165,000.00	831	Dominated
ET + CTA	R$ 255,572.95	884	
SE + CTA	R$ 325,477.75	909	
CTA	R$ 452,050.00	871	Dominated
CTA + ET	R$ 463,732.00	884	Dominated
CTA + SE	R$ 516,301.00	909	Dominated
CTA + MS	R$ 760,295.15	904	Dominated
MS	R$ 791,590.00	691	Dominated
MS + CTA	R$ 1,034,340.85	904	Dominated

*ET: exercise test; MS: myocardial scintigraphy; CTA: computed tomography angiography; SE: stress echocardiogram. Year 2020 as reference for the presented values.*

The four undominated strategies were listed in ascending order of costs, and it was identified that the ET + CTA strategy was the least effective (least number of correct diagnoses) and with a higher ICER than the SE + CTA strategy ( Table 4 ), and was thus not considered to be cost-effective (extended dominance).

**Table 4:** – Identification of extended dominance in the undominated diagnostic strategies for CAD

Strategy	Cost	Correct diagnosis	ICER	
ET	R$ 30,000.00	681.00	NA	
ET + SE	R$ 112,335.00	852.96	R$ 478.80	
ET + CTA	R$ 255,572.95	883.75	R$ 4,651.19	Extended dominance
SE + CTA	R$ 325,477.75	909.49	R$ 2,716.44	

*ET: exercise test; MS: myocardial scintigraphy; CTA: computed tomography angiography; SE: stress echocardiogram. Year 2020 as reference for the presented values; ICER: incremental costeffectiveness ratio.*

Thus, the efficiency frontier was constructed based on the three most cost-effective strategies, ET, ET+SE, and SE+CTA ( Figure 1 ).

**Figure 1:** – Efficiency frontier of diagnostic strategies for CAD (costs and number of correct diagnoses per 1,000 individuals). ET: exercise test; MS: myocardial scintigraphy; CTA: computed tomography angiography; SE: stress echocardiogram.

Based on the sensitivity analysis, shown in the Tornado Graph ( Figure 2 ), the parameters with greater impact on the results were sensitivity and specificity of the exercise test, the cost of the CTA, and the prevalence of CAD.

**Figure 2:** – Tornado Diagram – impact on the ICER values of each parameter evaluated separately in their upper and lower limits.

The NHB ranking enables the assessment of all of the strategies without the need to exclude dominated or extended dominance strategies. Table 5 presents the ranking of more cost-effective strategies according to the variation of pre-test probabilities between 10% and 60% in different thresholds of willingness to pay for a correct diagnosis, using the Brazilian GDP per capita as the base value, which, according to the most recent IBGE 2017 census is R$ 31,833.50. ^19^

**Table 5:** – Ranking of the diagnostic strategies by threshold of willingness to pay for correct diagnosis according to the net benefit criteria, in relation to the different pre-test probabilities

	0.05 GDP pc	0.1 GDP pc	0.2 GDP pc	0.3 GDP pc	0.4 GDP pc	0.5 GDP pc	1 GDP pc
10%	ET + SE	ET + SE	SE + CTA	SE + CTA	SE + CTA	SE + CTA	SE + CTA
20%	ET + SE	ET + SE	SE + CTA	SE + CTA	SE + CTA	SE + CTA	SE + CTA
30%	ET + SE	CTA + ET	SE + CTA	SE + CTA	SE + CTA	SE + CTA	SE + CTA
40%	ET + SE	CTA + ET	SE + CTA	SE + CTA	SE + CTA	SE + CTA	SE + CTA
50%	SE	CTA + ET	CTA	CTA	CTA	CTA	CTA
60%	SE	SE	CTA	CTA	CTA	CTA	CTA

*ET: exercise test; MS: myocardial scintigraphy; CTA: computed tomography angiography; SE: stress echocardiogram; GDP: gross domestic product.*

## Discussion

The SE + CTA strategy presents the best rate of correct diagnosis (909.49), in other words, the best effectiveness, and therefore the greatest certainty in the clinical guidance of the patients, be it for CA, or be it to eliminate the diagnosis. The definition of the best strategies to investigate obstructive CAD result in the best diagnostic certainty, thus minimizing the number of false-negatives (loss of diagnosis), as well as false-positives (reducing the number of “white” catheterisms and their complications). ^20 , 21^ Diagnostic errors in this scenario are associated with unnecessary invasive exams, in addition to leading to complications, such as acute myocardial infarction and death due to the lack of adequate treatment in a disease of high mortality. ^1 , 2 , 20^ The accuracy of the diagnostic tests should be analyzed in light of their costs, primarily when we consider a new technology in a publicly financed health system, such as SUS.

The ICER has been used by health technology assessment agencies worldwide to summarize the results of economic assessments of health interventions. Even in countries like Brazil, where there is no explicitly defined ICER threshold for decision-making, its impact on decisions is highly relevant. However, alternative resolute measures based on the concept of net benefit are being presented, and this is the first study to assess the cost-benefit of diagnostic strategies for CAD through an analysis of net benefit in health.

There are important distinctions between the ICER and the NHB. The ICER requires the comparison of two strategies, regardless of the total number of evaluated strategies. In the NHB, the net benefit measures are calculated for each strategy individually, that is, it eliminates the need to compare pairs and the need to eliminate dominated strategies. What is necessary is a defined threshold of the willingness to pay to calculate the measure of net benefits, but they are not necessary to calculate the ICER, although without a threshold of cost-effectiveness, its interpretation would be limited. No cost-effectiveness threshold has been established in Brazil in the process of the incorporation of technologies. In this study, the most cost effective strategy, according to the efficiency frontier, was the combination of the stress echocardiogram, followed by the computed tomography angiography, given that it is the undominated strategy with the highest ICER value and within a threshold.

The NHB criterion allows for the ranking of all of the strategies in such a way as to aid the decision-maker in choosing which strategy to follow, based on the availability of the exams/professionals, budget, and willingness to pay for a correct diagnosis. The CTA, although it is the only exam not included today in SUS, is the most prevalent exam among the most cost-effective strategies, only failing to reach first place when the willingness to pay is less than 0.2 GDP per capita per correct diagnosis. Separately, not considering sequential exams, the most cost-effective exam is the stress echocardiogram, up to the threshold of 0.1 GDP per capita per correct diagnosis, surpassed by the CTA in the upper thresholds.

When varied in different pre-test probabilities (10% to 60%), we find that the CTA is the most cost-effective test (combined or not with other methods) in 79% of the scenarios analyzed in this study, which is in accordance with cost-effectiveness studies of CTA conducted in developed countries ^20 - 23^ and with the recent updates from the UK’s *National Institute for Heath and Care Excellence* guidelines from 2017 ^22 , 24^ and the European Society of Cardiology (ESC) guidelines. ^25^ Updated in 2019, this guideline determines that the CTA can be used as the first-line exam in the evaluation of suggestive symptoms of obstructive CAD, substituting functional imaging exams. In the UK, which has a health system financed with public resources (like SUS in Brazil) based on cost-effectiveness analyses for their reality, the CTA was recommended as the first-line exam, substituting functional exams. ^22 , 24^ This decision should be taken based on the reality of each country and each health system, with the aim of the present study being to foster the understanding of the diagnostic strategies of thoracic pain within the reality of SUS.

The choice of diagnostic strategy should take into consideration not only the cost-effectiveness for the finding of the coronary obstruction, but also the clinical outcomes. Major randomized studies, such as PROMISE and SCOT HEART, have brought information mainly about the prognosis of those patients who began the CAD investigation with CTA. There was a greater certainty in the diagnosis and, with this, a greater introduction of preventive medicine therapies, considering a real possibility in the reduction of events, such as long-term infarctions, in addition to documenting a lesser number of CAs without obstructive disease in this group of patients. ^26 , 27^ In this context, it is important to highlight that the large randomized ISCHEMIA study failed to demonstrate any reduction in deaths or limb infarctions in the functional exams. ^28^ By contrast, the anatomical evaluation enables the diagnosis of atherosclerosis and refers the patient to a better clinical treatment, with the possibility of a reduction in infarctions and deaths. ^26 , 27 , 29^ The final guideline of the Brazilian Society of Cardiology (SBC) from 2014 recommends beginning the investigation of obstructive CAD with functional exams, followed by CTA should such exams be inconclusive or contraindicated. ^30^

Among the functional methods, the MS is one of the most commonly used tests in Brazil and worldwide to diagnose CAD. ^31 , 32^ It is estimated that, in SUS, approximately 54% of the elective nuclear medicine exams are of myocardial perfusion. ^32^ However, the strategies that include MS were dominated in our study, which proved to be the most expensive and the least effective.

The ET + SE and SE + CTA strategies present percentages of false-positives similar to 4% (Table 2:2, attached here), whereas the percentage of false-negatives varies considerably among the strategies. For example, the ET + SE strategy presents a false-negative rate of 1.3%, while the ET + CTA strategy presents a false-negative rate of 0.4% (three-fold less than the ET + SE), thus presenting less incorrect diagnoses. Although it has an extended dominance in the effectiveness frontier, in the analysis of the NHB, we observed that the ET + CTA strategy loses to the ET + SE strategy only within the margin of 0.05 to 0.1 GDP per capita (table NHB attached here). Asymmetric economic characteristics between regions and cities in Brazil make the availability of equipment and qualified workforce heterogeneous. For example, in 2019, among the nearly six million CT exams performed in SUS, 51% were concentrated in the Southeast region and less than 6% in the North. ^33^ The ranking of the diagnostic options presented in this work can aid decision-makers by combining local data on infrastructure, willingness to pay, and diagnostic accuracy. The CTA is less well-known and makes use of a more expensive machinery than does the SE, which may well limit its use as a first-line exam in scenarios with a lower budget, in which the ideal would be to begin with a lower cost and less complex exam, with the results used as referrals for more expensive and complex exams. As the ET is more well-known than the SE, one should consider that the ET + CTA strategy may well be more executable in the Brazilian public health system than would be the SE + CTA strategy, despite the minor drop in effectiveness from that of the ET as compared to that of the SE. The present study mainly analyzed patients in a 30% pre-test probability, as this is the prevalence of disease normally found in the outpatient diagnostic laboratories (considered mild-low). After performing the sensitivity tests, the results of cost-effectiveness do not reveal substantial changes when we vary the pre-test probability. In addition, the pre-test probability between 10% and 60% was ranked according to the willingness to pay, considering that the CTA was not found as a cost-effective option only when we considered the value of 0.05 GDP per capita per correct diagnosis.

Among the limitations of this study, discrepancies were found among the articles, mainly regarding the definition of obstructive CAD, in which the studies about CTA and MS define coronary obstruction as being above 50% by means of coronary angiography, while the study that includes exercise tests and stress echocardiogram includes articles with obstructive CAD references above 50% or 70%. To minimize possible biases, systematic reviews, meta-analyses, and quality analyses of the articles were performed. A recent cost-effectiveness study, with data from SUS, presented, as its main limitation, the determination of the CTA value as the cost paid by SUS for a simple chest CT, extrapolating data from the supplementary health system. ^34^ Our study attempts to reach the real value of the costs of CTAs (approximately 3 times the value of the chest CT), since this value has a major impact on the assessment of comparative strategies. Finally, the accuracy of the tests varies with the quality of the equipment and of the responsible staff. Future studies can verify the impact of the adoption of this flow chart of decision-making through the prospective follow-up by computing clinical and economic data from real world results.

## Conclusion

In scenarios like that of Brazil, with budget restrictions and heterogeneity in the supply of diagnostic tests, the identification of cost-effective strategies can guide health managers and decision-makers to manage their resources in a more efficient manner. Using up-to-date concepts of health technology assessments, this study provides a ranking for decision-making regarding which diagnostic strategy to use in a population with an intermediate pre-test risk for CAD. With a feasible cost estimate adopted for CTA, it can be concluded that the impact of including this to the list of diagnostic arsenal would represent a cost-effective strategy in most of the evaluated scenarios with broad variations in the willingness to pay.
